# Evaluation of the Structural, Biological, and Bone Induction Properties of Sol–Gel–Derived Lithium‐Doped 68S Bioactive Glass—An in Vitro Study on Human Dental Pulp Stem Cells

**DOI:** 10.1002/cre2.70139

**Published:** 2025-04-30

**Authors:** Pejman Janbaz, Faeze Behzadpour, Kiana Ghanadan

**Affiliations:** ^1^ Department of Oral and Maxillofacial Surgery, Faculty of Dentistry Qazvin University of Medical Sciences Qazvin Iran; ^2^ Department of pediatric, School of dentistry, Dental Research Center, Avicenna Institute of Clinical Sciences, Avicenna Health Research Institute Hamadan University of Medical Sciences Hamadan Iran; ^3^ Dental Caries Prevention Research Center Qazvin University of Medical Sciences Qazvin Iran; ^4^ Department of Operative Dentistry, Faculty of Dentistry Qazvin University of Medical Sciences Qazvin Iran

**Keywords:** bioactive glass, lithium, mineralization, osteoinduction, stem cells

## Abstract

**Objectives:**

Calcium silicate–based bioactive glass shows enhanced ion release capabilities and promotes the formation of hydroxyapatite (HA). This study aimed to synthesize a sol–gel–derived 68S bioactive glass (BAG) incorporating lithium (Li) and evaluate its structural, biological, and osteoinductive properties using human dental pulp stem cells (hDPSCs).

**Materials and Methods:**

Two types of 68S BAG were synthesized using the sol–gel method: one containing 5 mol.% lithium nitrate (BGLi5) and a lithium‐free control (BG). Structural characterization and HA formation were assessed using field emission scanning electron microscopy (FESEM) and Fourier‐transform infrared spectroscopy (FTIR) before and after immersion in simulated body fluid (SBF) on Days 1, 3, and 7. The dissolution rates of the specimens were evaluated using inductively coupled plasma atomic emission spectroscopy (ICP‐AES) and pH analysis. Biological activities were investigated through cell viability (MTT assay), alkaline phosphatase (ALP) enzyme activity, and alizarin red staining to assess mineralization. Additionally, the antimicrobial efficacy of the materials was tested against *Streptococcus mutans* (SM).

**Results:**

FTIR and FESEM analyses confirmed the formation of HA crystals in BGLi5 specimens by Day 3 and in BG specimens by Day 7. The MTT assay demonstrated enhanced cell viability in both BG and BGLi5 compared to the control group. ALP activity, a marker of cell differentiation, was significantly elevated in the BGLi5‐DM group by Day 14. Alizarin red staining on Day 21 revealed a marked increase in mineralization in both BG and BGLi5, with the BGLi5‐DM group showing the highest mineralization levels. Furthermore, both BG and BGLi5 demonstrated significant antimicrobial activity against *SM*.

**Conclusion:**

The sol–gel–derived 68S BAG containing 5 mol.% Li is a biocompatible material that enhances cell proliferation, differentiation, and mineralization. The combination of BGLi5 with differentiation‐specific culture medium synergistically promotes osteogenic differentiation and mineralization, making it a promising candidate for dental and bone tissue engineering applications.

## 1 Introduction

Dental caries is one of the most prevalent oral health issues, and if left untreated, it can result in irreversible damage to the dental pulp (Ahmed et al. 2020). A promising approach to managing dental caries involves dentin and pulp regeneration using human dental pulp–derived stem cells (HDPSCs) (Huang 2011; Park et al. 2025). HDPSCs show responsive behavior to bacterial, physical, and chemical stimuli, making them a valuable tool in regenerative dentistry (Zhang et al. 2020; Zhong et al. 2020).

The potential of HDPSCs in tissue engineering (TE) was first demonstrated by Gronthos et al. who observed the formation of dentin‐like structures when HDPSCs were implanted into the subcutaneous layer of immunocompromised mice. Since this finding was reported, HDPSCs have been widely utilized in TE applications (Gronthos et al. 2000).

Recent studies have highlighted the role of bioactive glass (BAG) in promoting the differentiation of HDPSCs and facilitating dentin repair through the formation of mineralized tissue (Ahn et al. 2020). BAG, composed of silicon (Si), calcium (Ca), and phosphorus oxides, is known for its biocompatibility, osteoinductive, and osteoconductive properties (Gong et al. 2014; Farano et al. 2019; Nandi et al. 2016; Vollenweider et al. 2007; Kavitha et al. 2014; Abbasi et al. 2015; Meskher et al. 2024; Degli Esposti et al. 2024). These characteristics are largely attributed to its ability to form hydroxyapatite (HA) (Pazhouheshgar et al. 2020; Zhang et al. 2019; Moghanian et al. 2017a), bind to vital structures (Zhang et al. 2019; Moghanian et al. 2017a; Pazhouheshgar et al. 2019), release ions (Zhang et al. 2019; Moghanian et al. 2017a; Pazhouheshgar et al. 2019; Saatchi et al. 2021), and show high surface reactivity (Moghanian et al. 2020a). Since its introduction to dentistry in 1971 by Hench et al (Abbasi et al. 2015). BAG has been used in various applications, including the treatment of periodontitis, bone lesions, implant coatings, mineralization, and even as an additive in toothpaste (Gong et al. 2014; Nandi et al. 2016; Karakuzu‐Ikizler et al. 2020). Among the various types of BAGs used in dentistry, orthopedics, and TE, the 68S, 58S, and 77S compositions are the most widely used (Moghanian et al. 2021). Specifically, 68S BAG, with a chemical composition of 70% SiO₂, 26% CaO, and 4% P₂O₅, is particularly suitable for tooth structure regeneration. Furthermore, the incorporation of modifiers such as aluminum (Al), calcium (Ca), copper (Cu), zinc (Zn), strontium (Sr), and lithium (Li) can enhance its degradability, bioactivity, and antibacterial properties (Omar et al. 2020; Chou et al. 2018; Moghanian et al. 2020a).

BAG can be synthesized using two primary methods: the melting method and the sol–gel method (Moghanian et al. 2020b). The sol–gel technique is often preferred over the melting method due to its superior control over homogeneity, the ability to create nanoporous structures, the ease of incorporating various ions into the glass matrix, and its lower synthesis temperatures. Additionally, sol–gel–derived BAGs show faster hydroxyapatite crystal formation and enhanced bioactivity, which has led to their increased adoption in recent years (Farano et al. 2019; Nandi et al. 2016; Moghanian et al. 2020b; Kermani et al. 2020; Zohourfazeli et al. 2021).

Previous studies have demonstrated the stimulatory effects of lithium (Li) on osteoblast proliferation and dentin regeneration (Kavitha et al. 2014; Cai et al. 2015; Khorami et al. 2011). Additionally, Li has been reported to play a role in immune system modulation, show antibacterial properties (Devi Balakrishnan et al. 2023) and, notably, contribute to a reduction in dental caries prevalence (Zhang et al. 2019; Saleh and El‐Adham 2018). In a 2017 study, Moghanian et al. investigated various compositions of 58S bioactive glass (BAG) containing Li at molar percentages ranging from 0% to 10%. They found that the composition with 5 mol.% Li showed the most favorable outcomes in terms of cell proliferation, differentiation, bioactivity, and antibacterial efficacy against methicillin‐resistant *Staphylococcus aureus* (MRSA) (Moghanian et al. 2017b). Given its extensive medical history, Li is considered a promising modifier for bioactive glasses (BAGs) (Cai et al. 2015). In summary, BAGs are widely recognized in modern science as biomaterials capable of promoting hard tissue regeneration, and the role of Li in dentin regeneration has been well documented (Khorami et al. 2011).

In the present study, a 68S BAG with a base chemical composition of 70% SiO₂, 26% CaO, and 4% P₂O₅ (molar percentages) was selected as the foundation for synthesizing and evaluating the effects of substituting 5 mol.% Li into the glass composition (BGLi5). The structural characteristics, bioactivity, antibacterial properties, and the proliferation and differentiation of human dental pulp stem cells (HDPSCs) in the presence of the modified BAG were systematically investigated.

## 2 Materials and Methods

### 2.1 Bioactive Glass (BAG) Synthesis

Bioactive glass (BAG) was synthesized using the sol–gel method. The raw materials included tetraethyl orthosilicate (TEOS, Merck), triethyl phosphate (TEP, Merck), calcium nitrate tetrahydrate (Ca(NO₃)₂·4H₂O, Merck), nitric acid (HNO₃, Merck), lithium nitrate (LiNO₃, Merck), and double‐distilled water. All chemicals were used as received, without further purification or additives.

### 2.2 Synthesis of Lithium‐Doped Bioactive Glass

The synthesis process began by mixing 2 M nitric acid with TEOS, followed by magnetic stirring for 30 min. Subsequently, TEP, Ca(NO₃)₂·4H₂O, and LiNO₃ were sequentially added to the solution. Stirring was continued until a clear sol was obtained (approximately 45 min). After the final addition, the solution was stirred for an additional hour to ensure complete hydrolysis.

The resulting sol was aged in sealed containers at 37°C for 3 days. The aged gel was then manually crushed using a laboratory spatula and dried at 75°C for 3 days. The dried gel was ground in a planetary ball mill using aluminum balls for 10 min at 200 rpm, with a ball‐to‐powder weight ratio of 10:1. The powder was subsequently stabilized at 700°C and re‐milled.

For tablet preparation, 0.31 g of the synthesized powder was compressed using a hydraulic press (Carver, Model 3912) and a custom mold to produce tablets with a diameter of 12 mm. The tablets were immersed in 30 mL of simulated body fluid (SBF, Aperin Advanced Technology Development Company) for 1, 3, and 7 days. Two types of tablets were prepared: lithium‐doped bioactive glass (BGLi5) and lithium‐free bioactive glass (BG). A control specimen was also prepared, which was not exposed to SBF.

### 2.3 Characterization of the BAG Surface and SBF Solution

The formation of a hydroxyapatite (HA) layer on the surface of bioactive glass (BG) was investigated using field emission scanning electron microscopy (FESEM) (Mira3‐XMU) and Fourier‐transform infrared spectroscopy (FTIR) (NICOLET 800). Before FESEM analysis, the specimens were coated with a thin layer of gold. FTIR spectra were analyzed within the wavelength range of 4000–400 cm⁻¹. Changes in ion concentrations within the simulated body fluid (SBF) solution were assessed using inductively coupled plasma atomic emission spectroscopy (ICP‐AES). Additionally, the pH of the SBF solution was monitored using a standard pH meter (Corning pH meter 340). Changes in ion concentrations and pH levels provide critical insights into the mechanisms underlying HA layer formation.

### 2.4 Biological Studies

#### 2.4.1 Cell Culture

Human dental pulp stem cells (HDPSCs) were obtained from a cell bank (Stem cell research center, Tehran, Iran). These cells were originally isolated from freshly extracted third molars of healthy donors (aged 18–25 years) after informed consent was obtained. The dental pulp tissue was carefully dissected, minced into small fragments, and enzymatically digested using 3 mg/mL collagenase type I (Sigma‐Aldrich, USA) for 1 h at 37°C. The digested tissue was filtered through a 70 µm cell strainer, and the resulting cell suspension was centrifuged at 1200 rpm for 5 min. The cell pellet was resuspended in Dulbecco's Modified Eagle's Medium (DMEM) (Gibco, USA) supplemented with 10% fetal bovine serum (FBS), 1% penicillin‐streptomycin, and a 1% antimycotic solution. The cells were cultured in a humidified incubator at 37°C with 5% CO₂ until they reached 80–90% confluence. Cell viability was assessed using trypan blue staining, and cell counting was performed using a Neubauer chamber. The average cell count was calculated using the standard formula for cell density determination.

The total number of cells in the Falcon flask was determined using the formula

$$\mathrm{Total}\;\mathrm{cell}\;\mathrm{count}=\mathrm{Falcon}\;\mathrm{volume}\times 10^{4}\times 2\times \mathrm{average}\;\mathrm{cell}\;\mathrm{count}.$$

The required volume of fresh medium was calculated to achieve the optimal cell density for culture. The old medium was removed, and the cells were washed with phosphate‐buffered saline (PBS) before adding fresh culture medium supplemented with 10% fetal bovine serum.

An indirect method was used to study the effects of bioactive glasses (BAGs) on HDPSCs. The BAGs were sterilized using ultraviolet light and then added to DMEM medium under sterile conditions in a cell culture hood. To release the active elements of the BAGs into the culture medium, the BAGs were immersed in the medium before cell culture. The immersion duration varied according to the study group (1, 3, 7, 14, and 21 days). The medium was filtered through a 0.2 µm membrane to remove potential microbial contaminants and residual BAG particles (Anesi et al. 2020). The prepared medium was then added to the HDPSC culture for subsequent biological studies (Gentleman et al. 2010).

#### 2.4.2 MTT Assay

The 3‐(4,5‐dimethylthiazol‐2‐yl)‐2,5‐diphenyltetrazolium bromide (MTT) assay was used to evaluate the cytotoxicity of the synthesized BAGs. HDPSCs were cultured in a 96‐well plate (Sigma‐Aldrich, USA). Prepared solutions of BG and BGLi5 (0.5, 1, 2.5, and 5 mg/mL) were added to the wells and incubated at 37°C with 5% CO₂. On days 1, 3, and 7, the MTT reagent (Sigma‐Aldrich, USA) was added to each well at a concentration of 0.45 mg/mL and incubated for 4 h at 37°C. The formazan crystals formed in the mitochondria were solubilized using dimethyl sulfoxide (DMSO) (Sigma‐Aldrich, USA). After 15 s of gentle agitation, the absorbance was measured at 570 nm using a spectrophotometer (Moonesi Rad et al. 2018).

#### 2.4.3 Alkaline Phosphatase (ALP) Enzyme Activity

The activity of Alkaline Phosphatase (ALP) was measured to assess the differentiation potential of Human Dental Pulp Stem Cells (HDPSCs). The experiment involved seeding HDPSCs into 96‐well ALP assay plates, which were divided into five experimental groups. The plates were incubated for 24 h at 37°C under 5% CO₂. The groups were defined as follows:

1. **BG‐M**: DMEM culture medium containing lithium‐free bioactive glass (BG).
2. **BGLi5‐M**: DMEM culture medium containing lithium‐substituted BG.
3. **Differentiation Medium (DM)**: Osteogenic differentiation medium (Osteoplus, Sigma Aldrich, USA) composed of high‐glucose DMEM, 5% fetal bovine serum (FBS), dexamethasone, beta‐glycerol phosphate, and ascorbic acid bisphosphate, without BG.
4. **BG‐DM**: Osteogenic differentiation medium containing lithium‐free BG.
5. **BGLi5‐DM**: Osteogenic differentiation medium containing lithium‐substituted BG.

The optimal concentrations of BG and BGLi5 (2.5 and 5 mg/mL, respectively) were determined using the MTT assay, ensuring minimal cytotoxicity and maximal bioactivity after 24 h of incubation. The culture medium was refreshed every other day until the designated time points. On Days 7, 14, and 21, the medium was removed, and the cells were washed with phosphate‐buffered saline (PBS). Subsequently, 500 µL of cold RIPA buffer (containing NaCl, EDTA, Tris, and dH₂O; Sigma Aldrich, USA) was added to each well. After 10 min, the lysates were collected, centrifuged at 4°C (1200 rpm, 5 min), and the supernatants were transferred to new plates.

For the ALP assay, solutions R1 and R2 from the ALP kit were mixed in a 10:1 ratio and added to the wells. The compositions of the solutions were as follows:

- **R1**: 1.0 mol/L diethanolamine and 0.5 mmol/L magnesium chloride.
- **R2**: 10 mmol/L p‐nitrophenylphosphate.

The plates were incubated for 1.5 h at 37°C, and ALP activity was quantified using a spectrophotometer at an optical density (OD) of 405 nm.

#### 2.4.4 Alizarin‐Red Staining

Alizarin‐Red staining was used to assess the calcification induced by differentiated cells. Six experimental groups were defined, including those used in the ALP enzyme activity assay, along with an additional control group designated as the medium (M) group, which consisted of DMEM culture medium without bioactive glass (BG). The groups were cultured in 96‐well plates for Alizarin‐Red staining. On Day 21, the culture medium was removed, and the samples were washed with phosphate‐buffered saline (PBS). Subsequently, 4% paraformaldehyde (Sigma Aldrich, USA) was added, and the plates were refrigerated for 20 min. After returning to room temperature for 5 min, the paraformaldehyde was removed and the samples were rinsed with PBS.

A 2% Alizarin‐Red staining solution (Sigma Aldrich, USA) was prepared by dissolving 2 g of the dye in 100 mL of distilled water, and the pH was adjusted to 4.1–4.3 using 0.5% NaOH. The staining solution (300 µL) was added to each well and incubated at room temperature for 3–4 min. Excess dye was removed by rinsing with PBS and paraformaldehyde. Images of the stained plates were captured using a microscope (Huang et al. 2016). Quantification of Alizarin‐Red staining was performed following the methodology described by Gutiérrez et al. (2012). ImageJ software was utilized for image digitization and processing. Threshold adjustment was applied to identify red‐stained areas, and the results were expressed as a percentage of the total area (Gutiérrez et al. 2012).

#### 2.4.5 Antibacterial Studies

The antibacterial efficacy of the synthesized BGs was evaluated against *Streptococcus mutans* (SM), a highly cariogenic pathogen implicated in oral health deterioration. SM (ATCC 25175) was cultured on brain–heart infusion (BHI) agar medium for 24 h at 37°C under 5% CO₂. A homogeneous bacterial suspension was prepared in distilled water, and the bacterial concentration was adjusted to 100,000 CFU/mL. The bacterial suspension was inoculated into BHI broth containing BGs at a concentration of 10,000 ppm. A control group, consisting of BG‐free BHI broth, was also prepared.

The cultures were incubated in a shaker incubator at 37°C and 5% CO₂ for 24 h. After incubation, the suspensions were serially diluted, and 200 µL of each dilution was plated onto BHI agar plates. The plates were incubated for an additional 24 h under the same conditions. Colonies were counted on plates containing 30–300 CFUs. The bacterial survival rate was calculated using the following formula:

Survivalrate(%)=[1−(Numberofsurvivedbacteriainthestudygroup/Numberofsurvivedbacteriainthecontrolgroup)]×100.

### 2.5 Statistical Analysis

Data analysis was conducted using GraphPad Prism software. Normality was assessed using the Kolmogorov–Smirnov test. For comparisons between groups, the *t*‐test, one‐way ANOVA, and Tukey's post hoc tests were used as appropriate. A probability value of less than 0.05 (*p* < 0.05) was considered statistically significant.

## 3 Results and Discussion

### 3.1 FESEM Analysis

**Figure 1:**
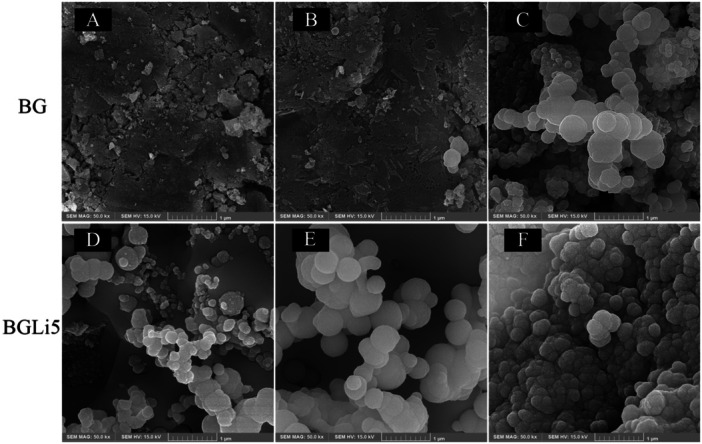
FESEM imaging of the BG on Day 1 of immersion (A), on Day 3 of immersion (B), on Day 7 of immersion (C), and BGLi5 on Day 1 of immersion (D), on Day 3 of immersion (E), and on Day 7 of immersion (F).

The formation of globular hydroxyapatite (HA) on the surface of the specimens increased progressively from Day 1 to Day 7 (Figure 1A–E). Spherical HA particles were observed in the BGLi5 specimens starting from Day 3, whereas in the BG specimens, they became visible only by Day 7. This indicates that the formation of HA crystals occurred earlier in the BGLi5 group. Furthermore, the size and quantity of HA particles formed in the BGLi5 group were significantly greater than those in the BG group. These results demonstrate that the incorporation of the Li component into the bioactive glass (BAG) accelerates the rate of HA formation, which is consistent with the trends observed in the FTIR analysis.

### 3.2 FTIR Analysis

**Figure 2:**
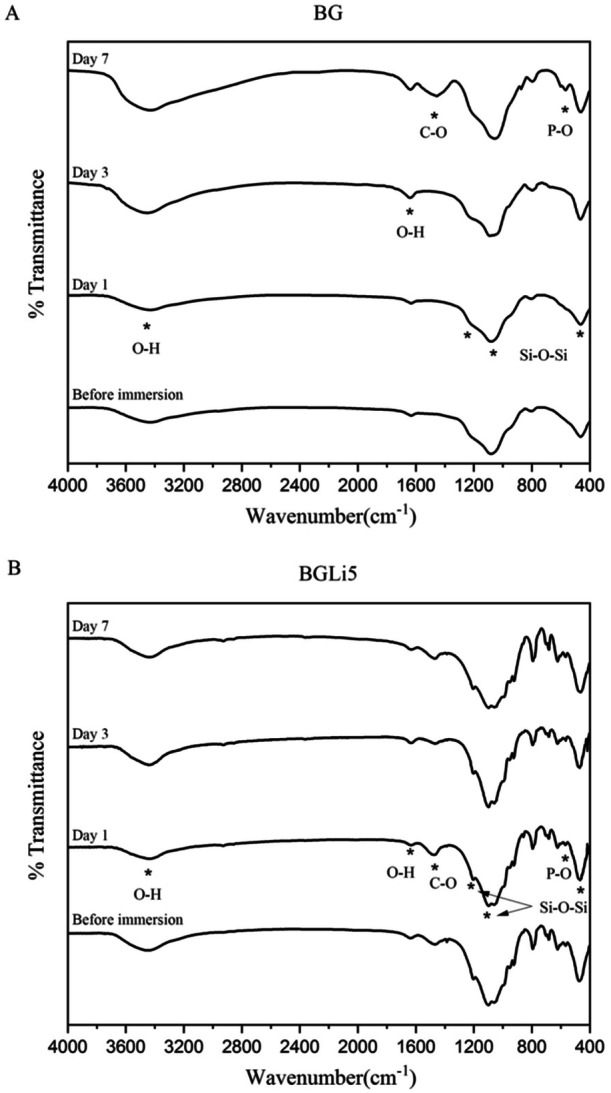
Infrared Fourier transform spectroscopy diagrams (FTIR) of BG specimens (A) and BGLi5 specimens (B) before and after immersion in the SBF solution on Days 1, 3, and 7.

The FTIR spectra of the synthesized bioactive glasses (BAGs) before and after immersion in simulated body fluid (SBF) on Days 1, 3, and 7 are presented in Figure 2. The FTIR spectra confirmed the accuracy of the chemical composition and stoichiometry of the synthesized specimens. Before immersion in SBF, the presence of Si–O–Si tensile and flexural bands in the FTIR spectra of all specimens indicated a high silicon content in the glass composition, consistent with previous studies (Wu et al. 2015). Similar peaks have been reported by Mozaffari et al. in the FTIR spectra of BAGs with the chemical composition SiO₂–CaO–P₂O₅ (Mozafari et al. 2010).

The emergence of P–O and C–O peaks in the spectra confirmed the formation of calcium phosphate and the subsequent development of an HA layer (Zhao et al. 2011). The intensity of these peaks increased over time in the BGLi5 group, reaching its maximum by Day 7, indicating extensive HA layer formation. In contrast, the BG group showed peaks associated with HA formation only by Day 7, suggesting a delayed process. These FTIR findings were further supported by the results of the FESEM analysis.

### 3.3 Inductively Coupled Plasma (ICP) Analysis

**Figure 3:**
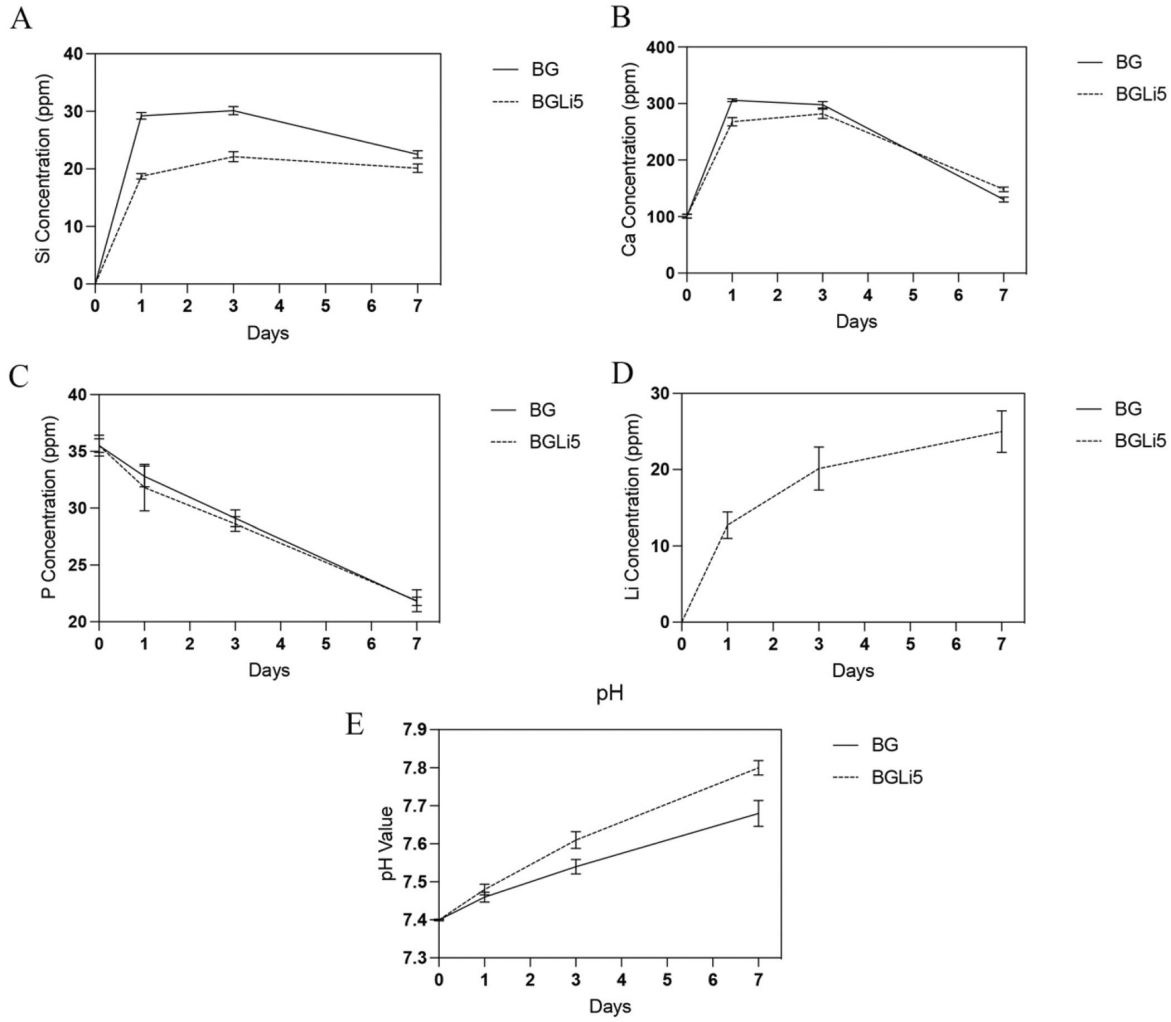
Silicon (A), Calcium (B), Phosphorus (C), and Lithium (D) ions' release and pH changes (E) of the BG and BGLi5 specimens in SBF solution.

The concentrations of Si, Ca, P, and Li ions, as well as changes in pH, were measured before and after specimen immersion in simulated body fluid (SBF) solution on Days 1, 3, and 7. The results are illustrated in Figure 3A–E.

An increase in Ca and Li ion concentrations, along with an increase in solution pH on the first day, suggests ion exchange between the bioactive glasses (BAGs) and the SBF solution. The Ca ion concentration was regulated similarly to the P ion concentration in the SBF solution, influenced by the release of ions from the BAG specimens and the formation of a hydroxyapatite (HA) layer. By the third day, the Ca ion concentration decreased, likely due to the rapid growth of apatite nuclei on the specimen surface, a finding corroborated by FTIR analysis.

The BGLi5 group showed a lower Ca release rate compared to the BG group, which can be attributed to the smaller atomic radius of Li. This results in higher oxygen density within the BGLi5 structure, strengthening lattice and oxygen bonds, thereby enhancing lattice stability and reducing Ca release. The simultaneous release and deposition of Li on the BAG surface, facilitated by its small atomic radius, created heterogeneous regions that promoted and accelerated HA formation (Brückner et al. 2016). Additionally, the higher oxygen density and lower Ca concentration in BGLi5 specimens contributed to the reduced Ca release rate. However, rapid cation exchange between Ca and H in the SBF solution led to the formation of hydroxyl groups, which are critical for the development of hydroxyl‐carbonate apatite (HCA) (Andersson and Kangasniemi 1991).

Si ions were detected in the SBF solution, with their concentration increasing up to the third day of the experiment. As Si is not present in the SBF solution, its release is attributed to BAG dissolution. As shown in Figure 3A, changes in the Si concentration mirrored those of Ca. The ion exchange between H from the SBF solution and Ca, Si, and Li from the BAGs increased the permeability of the glass structure, initiating Si release and the formation of silanol groups. Consequently, substituting Ca with Li in the BGLi5 network reduced Ca release, decreased permeability, and slowed Si release (Moghanian et al. 2018).

The P ion concentration in the SBF solution decreased steadily from Day 1 to Day 7, primarily due to HA formation, as evidenced by the consumption of available P ions (Figure 3B) (Zhao et al. 2011). According to Hench's theory, the formation of silanol (Si–OH) groups on the BAG surface is a critical step for HA formation in silica‐based glasses. These silanol groups create a negative surface potential, which facilitates Ca binding to the glass surface. Recent studies have confirmed that silica‐based glasses can generate sufficient Si–OH groups on their surface, enhancing HA phase formation during immersion in SBF solution (Arcos et al. 2003).

### 3.4 In Vitro Biological Evaluation

#### 3.4.1 MTT Assay

**Figure 4:**
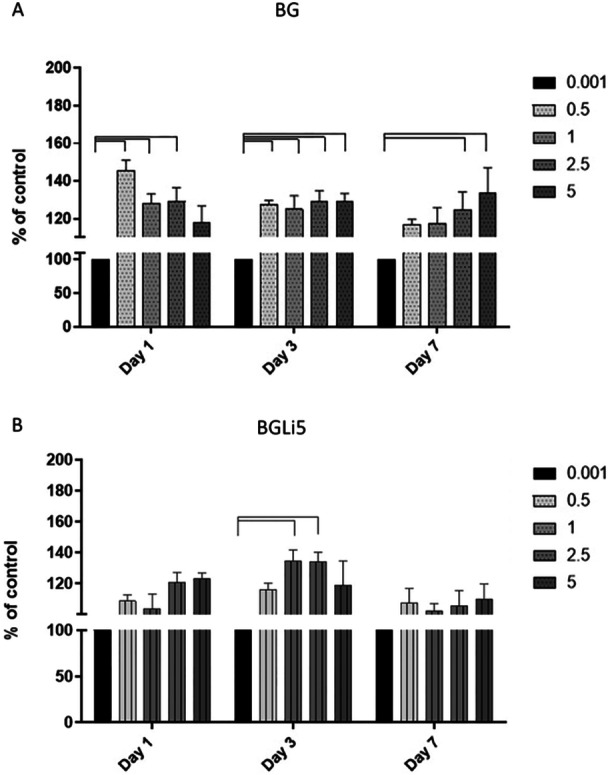
(A) MTT test results of BG and B) MTT test results of BGLi5 on Days 1, 3, and 7, using the following concentrations: 0.5, 1, 2.5, and 5 mg/mL. Each column represents a concentration. The black column represents the viability of the cells in the control group (TCP 0.001). The connection lines show a statistically significant difference between the mentioned groups (*p* < 0.05).

The results of the MTT assay, presented in Figure 4, were used to determine the optimal dose and confirm the non‐toxicity of the bioactive glass (BAG) specimens. The findings demonstrated that cell viability in both the BG and BGLi5 groups increased compared to the control group (TCP = 0.001). The non‐toxicity of the synthesized BAGs, irrespective of the lithium (Li) content, was confirmed in accordance with ISO 10993 standards. This aligns with previous studies carried out by Khorami et al. who evaluated 45S BAG containing 0%–12% Li₂O, and Tavakolizadeh et al. who investigated various BAG compositions, including 45S, 58S, and 63S (Khorami et al. 2011; Tavakolizadeh et al. 2017). According to ISO 10993, a substance is considered nontoxic if cell viability in its presence remains above 70% (Naruphontjirakul et al. 2019).

In the BGLi5 groups, concentrations of 1 and 2.5 mg/mL enhanced human dental pulp stem cell (HDPSC) survival on Day 3, but did not significantly affect proliferation by Day 7. This observation can be attributed to the initial burst release of lithium ions (Li⁺) from the bioactive glass. At these concentrations, the released Li⁺ may have temporarily supported cell survival on Day 3. However, by Day 7, the Li⁺ concentration likely reached a threshold where it no longer promoted proliferation, potentially due to the saturation of cellular signaling pathways or the initiation of differentiation processes. This is consistent with prior studies indicating that lithium ions can initially enhance cell survival but may not sustain proliferation over extended periods.

In the BG groups, dose‐ and time‐dependent variations in HDPSC proliferation were observed. At lower concentrations (0.5 mg/mL), the ions released from the bioactive glass provided an optimal environment for cell survival and initial proliferation on Day 1. However, at higher concentrations (5 mg/mL), the sustained release of ions over time created a more favorable microenvironment for cell proliferation by Day 7. This phenomenon is supported by previous studies demonstrating that higher concentrations of bioactive glass can enhance cell proliferation over longer durations due to the gradual release of bioactive ions such as Ca²⁺ and Si⁴⁺.

The MTT data revealed that cell proliferation increased until Day 3, before declining by Day 7. This finding contrasts with a similar study carried out by Khorami et al. (2011)., which may be explained by differences in cell lines. Although the present study utilized HDPSCs, Khorami et al. used osteoblast cells. The observed decline in proliferation by Day 3 may indicate the initiation of stem cell differentiation (Moayeri et al. 2017). Silicon (Si) ions were identified as a critical factor influencing cell viability. Inductively coupled plasma (ICP) analysis revealed that the Si concentration peaked on Day 3 and subsequently decreased by Day 7, consistent with the MTT results (Chou et al. 2014).

Based on the MTT findings, the optimal concentrations for further evaluation were determined to be 2.5 mg/mL for BGLi5 and 5 mg/mL for BG, as these doses were effective while minimizing potential toxicity.

#### 3.4.2 Alkaline Phosphatase Assay (ALP)

**Figure 5:**
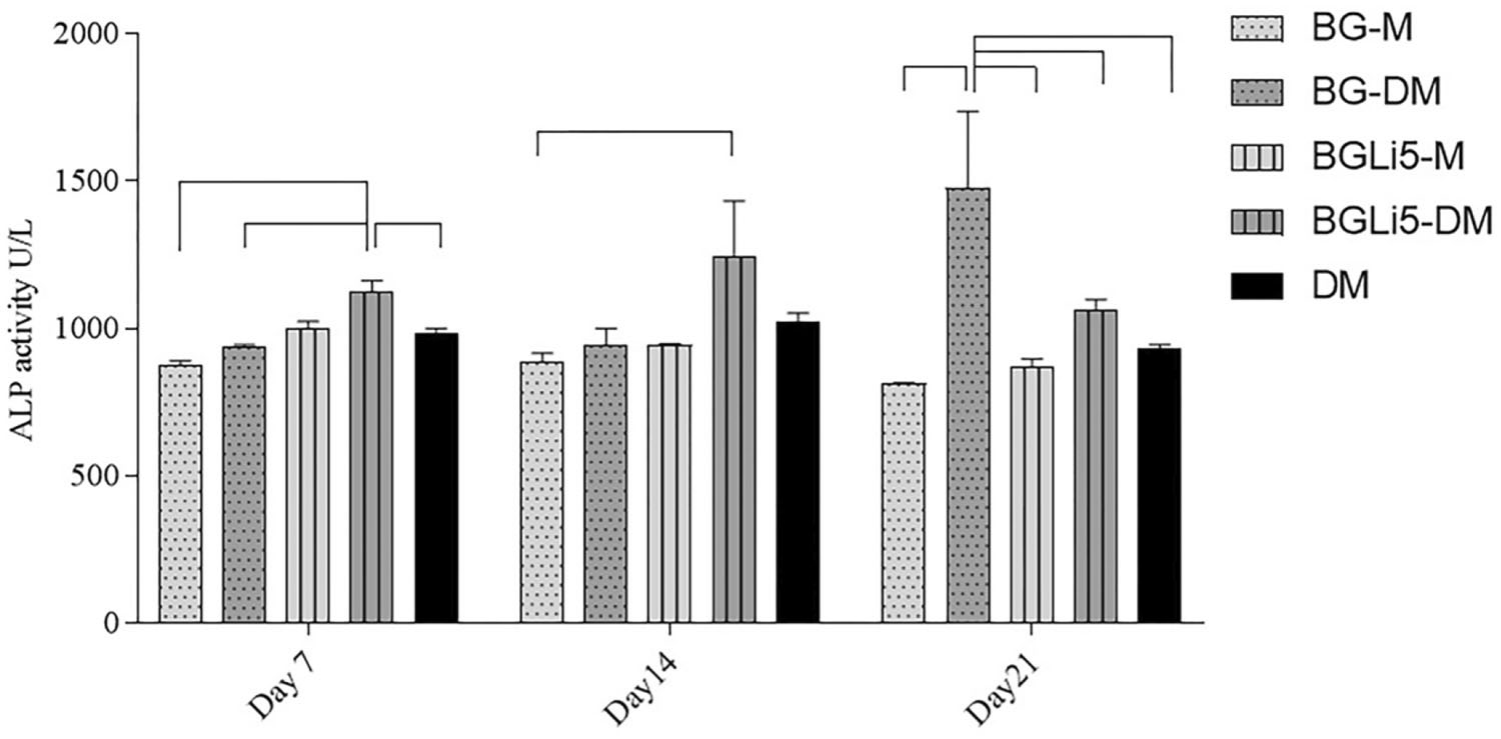
ALP activities on Days 7, 14, and 21 in BG‐M (BG in DMEM medium), BG‐DM (BG in differential medium), BGLi5‐M (BGLi5 in DMEM medium), BGLi5‐DM (BGLi5 in differential medium), and DM (BG free differential medium) groups. The connection lines show a statistically significant difference between the mentioned groups (*p* < 0.05).

The results of the ALP assay are presented in Figure 5. The assay was conducted using optimal doses determined by the MTT test. On Day 7 of the experiment, ALP levels in the BGLi5‐DM group showed a significant increase compared to the DM and BGLi5‐M groups. In the presence of differentiation‐inducing factors, such as dexamethasone and ascorbic acid, lithium (Li) showed a synergistic and enhancing effect on ALP activity. This aligns with prior studies demonstrating that dexamethasone promotes ALP activity and mineralization (Ciraldo et al. 2019; Hoemann et al. 2009). By Day 14, ALP levels increased across all groups, though these changes were not statistically significant. The highest increase was observed in the BGLi5‐DM group, consistent with the findings reported by Khorami (Khorami et al. 2011) and Tavakolizadeh (Tavakolizadeh et al. 2017).

From Day 14 to Day 21, ALP enzyme activity declined in all groups, except BG‐DM. This decrease aligns with established mechanisms of cell differentiation and mineralization, where a reduction in ALP activity corresponds to an increase in intracellular calcium, marking the transition from the initial differentiation phase to the mineralization phase (Hoemann et al. 2009). The presence of mineralized particles was further confirmed and quantified using Alizarin‐red staining on Day 21 (Yu et al. 2007).

Although previous studies on bioactive glasses (BAGs) measured ALP activity only up to Day 14 (Khorami et al. 2011; Tavakolizadeh et al. 2017), the present study extended observations to Day 21, providing additional insights into the temporal dynamics of ALP activity and mineralization. Consequently, direct comparisons with earlier findings are limited due to differences in experimental timelines.

#### 3.4.3 Alizarin‐Red Assay

**Figure 6:**
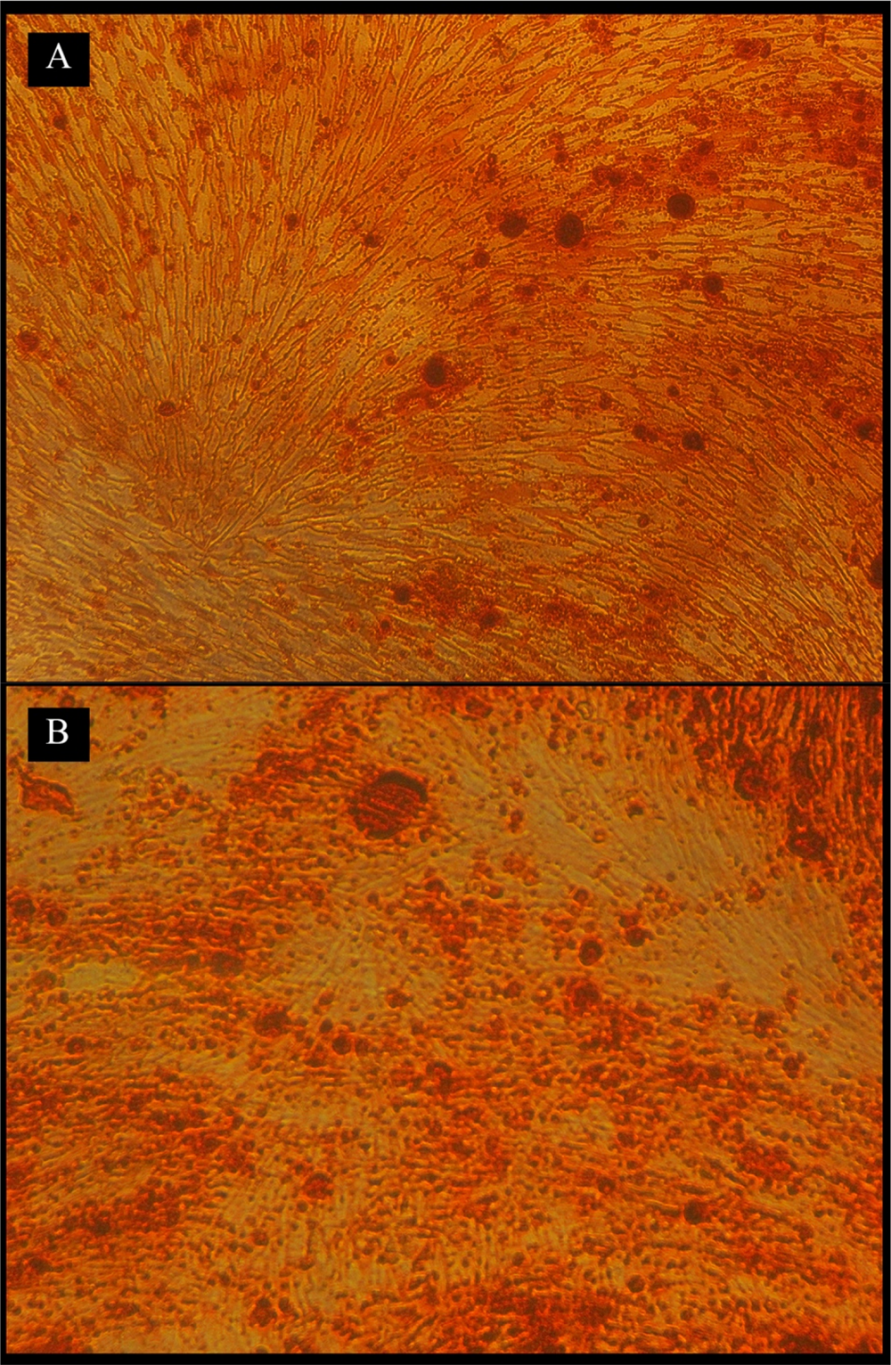
Alizarin‐Red staining of BG (A) and BGLi5 (B) specimens on Day 21.

**Figure 7:**
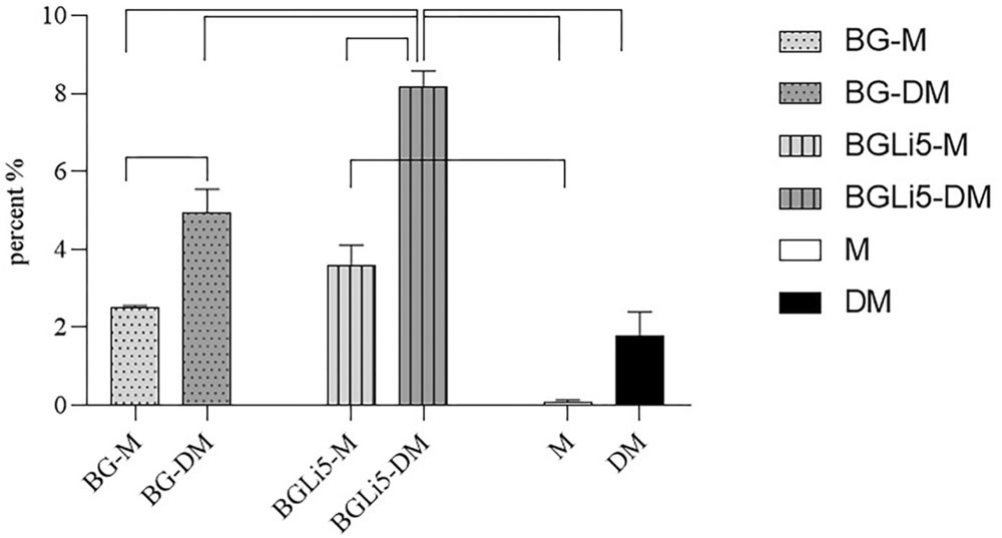
Alizarin‐Red staining results on Day 21 in BG‐M (BG in DMEM medium), BG‐DM (BG in differential medium), BGLi5‐M (BGLi5 in DMEM medium), BGLi5‐DM (BGLi5 in differential medium), DM (Pure BG free differential medium), and M (Pure DMEM culture medium) groups. The connection lines show a statistically significant difference between the mentioned groups (*p* < 0.05).

Alizarin‐red staining performed on Day 21 (Figure 6) demonstrated a significant increase in mineralized particle formation in the BGLi5‐DM group compared to other experimental groups (Figure 7). These findings align with the results of the ALP assay, further supporting the positive influence of lithium‐doped bioactive glass (Li‐doped BAG) on the differentiation and mineralization of human dental pulp stem cells (HDPSCs). The incorporation of lithium into the BAG structure not only enhanced its physical and structural properties but also improved its biological performance, particularly in promoting cell differentiation and mineralization. Previous studies have reported that lithium compounds can increase bone mineral density (Zamani et al. 2009; Tang et al. 2015); however, no prior research has specifically investigated the effects of Li‐doped BAG on mineralization processes.

The significant difference observed between the BGLi5‐DM and BGLi5‐M groups highlights the critical role of differentiation factors present in the differentiation‐specific culture medium (Hoemann et al. 2009). Furthermore, when compared to the DM group, the results confirm a synergistic effect between BGLi5 and the differentiation factors, enhancing mineralization outcomes.

Due to the lack of reported data on BAG‐mediated mineralization at Day 21 of culture, direct comparisons with existing studies were not feasible. Day 21 represents a critical time point in the osteogenic differentiation process, during which stem cells transition from a proliferative state to a fully differentiated state capable of forming mineralized particles.

Interestingly, although the ALP assay indicated that the BG group showed stronger osteogenic potential than the BGLi5 group on day 21, the ARS assay revealed that the BGLi5 group displayed superior osteoinductive capabilities. This apparent discrepancy can be attributed to the distinct stages of osteogenic differentiation assessed by the two assays. The ALP assay, which measures early osteogenic differentiation, showed higher activity in the BG group, suggesting that BG may more effectively initiate the differentiation process. In contrast, the ARS assay, which evaluates late‐stage mineralization, demonstrated that BGLi5 significantly enhanced mineralization, likely due to the sustained release of lithium ions, which are known to promote bone formation and mineralization.

Although MTT assays confirmed that both BG and BGLi5 supported HDPSC proliferation, variations in cell numbers among the control, BG, and BGLi5 groups on Day 21 could have influenced the ALP and ARS assay results. However, normalization of the assay data accounted for differences in cell density, ensuring that the observed effects on ALP activity and mineralization were attributable to the bioactive properties of the materials rather than variations in cell numbers. This normalization process underscores the distinct roles of BG and BGLi5 in promoting osteogenic differentiation and mineralization, respectively (Table 1).

**Table 1:** Elemental compositions of the synthesized BG and BGLi5 (mol. %).

Glass	Label	SiO_2_	CaO	P₂O₅	Li_2_O
68S‐0%Li_2_O	BG	60	36	4	0
68S‐5%Li_2_O	BGLi5	60	31	4	5

#### 3.4.4 Antimicrobial Study

**Figure 8:**
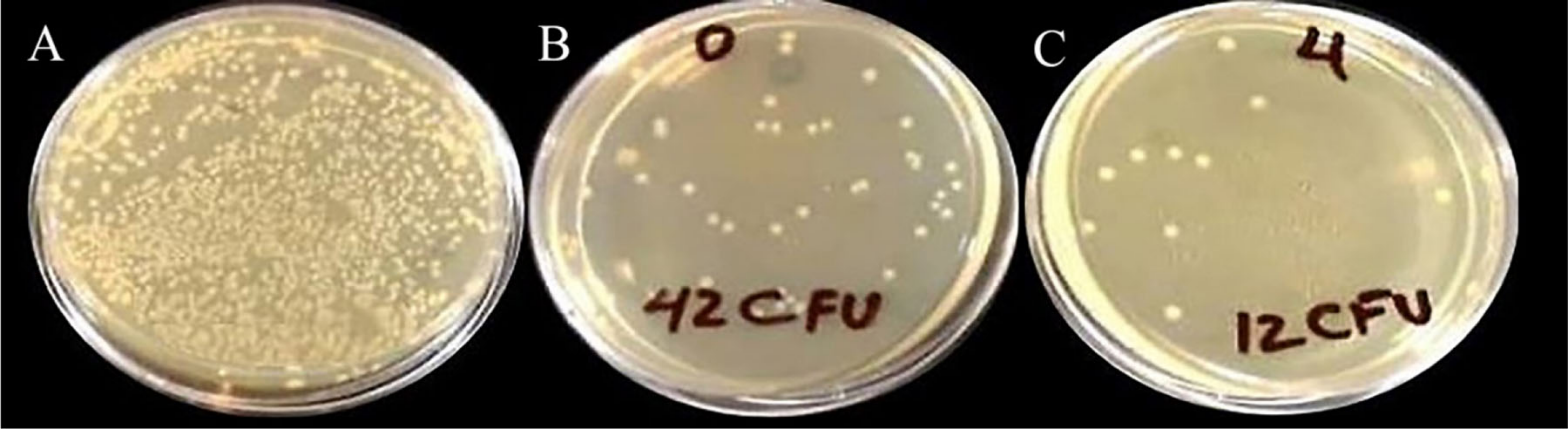
Antibacterial activity of BG and BGLi5 against streptococcus mutans. Control group (A), BG (B), and BGLi5 (C).

The mean reduction of bacterial colonies was compared between the study and control groups, as summarized in Table 2. The 68S bioactive glass (BAG) synthesized using the sol–gel method demonstrated significant inhibitory effects against SM bacteria (*p* < 0.01) (Figure 8). These findings align with previous research by Allan et al. who reported the antibacterial properties of BAGs against oral bacteria (Allan et al. 2001). In the present study, the synthesized BAG (BGLi5) showed notable antibacterial activity (*p* < 0.01). The incorporation of lithium (Li) nanoparticles into the BAG structure significantly enhanced its antibacterial efficacy (*p* < 0.01). Similar results were reported by Kavitha et al. who observed a substantial reduction in colony counts of various *Streptococcus* species upon adding 5% Li₂O to 58S BAG (Kavitha et al. 2014). Additionally, Nazemi et al. evaluated the antibacterial properties of 37S and 58S BAGs, noting that 37S BAG, with its higher CaO₂ content, demonstrated superior antibacterial activity over time. This was attributed to increased alkali oxide release, leading to elevated environmental pH levels (Nazemi et al. 2014). Begum et al. also reported comparable findings (Begum et al. 2016).

**Table 2:** Antibacterial activity (bacterial reduction rate) of BG and BGLi5 specimens against SM bacteria.

	BG	BGLi5
Mean ± SD (%)	99.58 ± 0.09	99.88 ± 0.06

In summary, the incorporation of antibacterial ions such as Li into BAGs can significantly enhance their physical and biological properties, including antibacterial activity.

## 4 Conclusion

The morphological, structural, biological, and antibacterial properties of sol–gel–derived SiO₂–CaO–P₂O₅–Li₂O bioactive glass containing 5 mol.% Li₂O (BGLi5) were comprehensively evaluated. Fourier‐transform infrared (FTIR) spectroscopy and field‐emission scanning electron microscopy (FESEM) confirmed the formation of hydroxyapatite on the surface of BGLi5 after 3 days of immersion in simulated body fluid (SBF). The MTT assay revealed increased cell viability after 1, 3, and 7 days of culture. Alkaline phosphatase (ALP) assay results indicated enhanced cell differentiation, particularly in the BGLi5‐DM group by day 14. Alizarin red staining on Day 21 further demonstrated a significant increase in mineralization foci. Antibacterial assessments showed a marked reduction in SM colony formation. The use of a differential culture medium further amplified mineralization, whereas the inclusion of Li in the BAG composition showed a synergistic effect, significantly accelerating the differentiation and mineralization of human dental pulp stem cells (HDPSCs).

## Author Contributions

Conceptualization: Kiana Ghanadan, Pejman Janbaz. Methodology: Kiana Ghanadan, faeze behzadpour, Pejman Janbaz. Formal analysis: Kiana Ghanadan, Pejman Janbaz. Writing – original draft preparation: Faeze Behzadpour. Writing – review and editing: Kiana Ghanadan, faeze behzadpour, and Pejman Janbaz. All authors have read and agreed to the published version of the manuscript.

## Conflicts of Interest

The authors declare no conflicts of interest.

## Data Availability

The data that support the findings of this study are available from the corresponding author upon reasonable request.

## References

[cre270139-bib-0001] Abbasi, Z. , M. Bahrololoom , M. Shariat , and R. Bagheri . 2015. “Bioactive Glasses in Dentistry: A Review.” Journal of Dental Biomaterials 2, no. 1: 1–9.

[cre270139-bib-0002] Ahmed, G. M. , E. A. Abouauf , N. AbuBakr , C. E. Dörfer , and K. F. El‐Sayed . 2020. “Tissue Engineering Approaches for Enamel, Dentin, and Pulp Regeneration: An Update.” Stem Cells International 2020, no. 1: 5734539. 32184832 10.1155/2020/5734539 PMC7060883

[cre270139-bib-0003] Ahn, J. H. , I.‐R. Kim , Y. Kim , et al. 2020. “The Effect of Mesoporous Bioactive Glass Nanoparticles/Graphene Oxide Composites on the Differentiation and Mineralization of Human Dental Pulp Stem Cells.” Nanomaterials 10, no. 4: 620. 32230907 10.3390/nano10040620 PMC7221817

[cre270139-bib-0004] Allan, I. , H. Newman , and M. Wilson . 2001. “Antibacterial Activity of Particulate Bioglass® Against Supra‐And Subgingival Bacteria.” Biomaterials 22, no. 12: 1683–1687. 11374470 10.1016/s0142-9612(00)00330-6

[cre270139-bib-0005] Andersson, Ö. H. , and I. Kangasniemi . 1991. “Calcium Phosphate Formation at the Surface of Bioactive Glass in Vitro.” Journal of Biomedical Materials Research 25, no. 8: 1019–1030. 1918106 10.1002/jbm.820250808

[cre270139-bib-0006] Anesi, A. , G. Malavasi , L. Chiarini , R. Salvatori , and G. Lusvardi . 2020. “Cell Proliferation to Evaluate Preliminarily the Presence of Enduring Self‐Regenerative Antioxidant Activity in Cerium Doped Bioactive Glasses.” Materials 13, no. 10: 2297. 32429291 10.3390/ma13102297 PMC7288167

[cre270139-bib-0007] Arcos, D. , D. C. Greenspan , and M. Vallet‐Regí . 2003. “A New Quantitative Method to Evaluate the in Vitro Bioactivity of Melt and Sol‐Gel‐Derived Silicate Glasses.” Journal of Biomedical Materials Research. Part A 65, no. 3: 344–351. 12746881 10.1002/jbm.a.10503

[cre270139-bib-0008] Begum, S. , W. E. Johnson , T. Worthington , and R. A. Martin . 2016. “The Influence of pH and Fluid Dynamics on the Antibacterial Efficacy of 45S5 Bioglass.” Biomedical Materials 11, no. 1: 015006. 26836582 10.1088/1748-6041/11/1/015006

[cre270139-bib-0009] Brückner, R. , M. Tylkowski , L. Hupa , and D. S. Brauer . 2016. “Controlling the Ion Release From Mixed Alkali Bioactive Glasses by Varying Modifier Ionic Radii and Molar Volume.” Journal of Materials Chemistry B 4, no. 18: 3121–3134. 32263050 10.1039/c5tb02426a

[cre270139-bib-0010] Cai, Y. , L. Guo , H. Shen , et al. 2015. “Degradability, Bioactivity, and Osteogenesis of Biocomposite Scaffolds of Lithium‐Containing Mesoporous Bioglass and mPEG‐PLGA‐b‐PLL Copolymer.” International Journal of Nanomedicine 10: 4125–4136. 26150718 10.2147/IJN.S82945 PMC4484672

[cre270139-bib-0011] Chou, M.‐Y. , C.‐T. Kao , C.‐J. Hung , et al. 2014. “Role of the P38 Pathway in Calcium Silicate Cement–Induced Cell Viability and Angiogenesis‐Related Proteins of Human Dental Pulp Cell in Vitro.” Journal of Endodontics 40, no. 6: 818–824. 24862709 10.1016/j.joen.2013.09.041

[cre270139-bib-0012] Chou, Y.‐J. , C.‐W. Hsiao , N.‐T. Tsou , M.‐H. Wu , and S.‐J. Shih . 2018. “Preparation and in Vitro Bioactivity of Micron‐Sized Bioactive Glass Particles Using Spray Drying Method.” Applied Sciences 9, no. 1: 19.

[cre270139-bib-0013] Ciraldo, F. E. , K. Schnepf , W. H. Goldmann , and A. R. Boccaccini . 2019. “Development and Characterization of Bioactive Glass Containing Composite Coatings With Ion Releasing Function for Antibiotic‐Free Antibacterial Surgical Sutures.” Materials 12, no. 3: 423. 30704083 10.3390/ma12030423 PMC6385048

[cre270139-bib-0014] Degli Esposti, L. , K. Zheng , A. Piancastelli , et al. 2024. “Composite Materials of Amorphous Calcium Phosphate and Bioactive Glass Nanoparticles for Preventive Dentistry.” Ceramics International 50, no. 1: 593–602.

[cre270139-bib-0015] Devi Balakrishnan, P. , N. P. Rath , T. Premkumar , A. Ganesh , and P. Kanchana . 2023. “Facile and Green Synthesis, Crystal Structure, Antibacterial Activity, Hirshfeld Surface Analysis, and Computational Investigation of a Novel Lithium (I) Complex: Comparisons of Theoretical and Experimental Analyses.” Journal of Molecular Liquids 391: 123118.

[cre270139-bib-0016] Farano, V. , J. C. Maurin , N. Attik , P. Jackson , B. Grosgogeat , and K. Gritsch . 2019. “Sol–Gel Bioglasses in Dental and Periodontal Regeneration: A Systematic Review.” Journal of Biomedical Materials Research, Part B: Applied Biomaterials 107, no. 4: 1210–1227. 30199601 10.1002/jbm.b.34214

[cre270139-bib-0017] Gentleman, E. , Y. C. Fredholm , G. Jell , et al. 2010. “The Effects of Strontium‐Substituted Bioactive Glasses on Osteoblasts and Osteoclasts in Vitro.” Biomaterials 31, no. 14: 3949–3956. 20170952 10.1016/j.biomaterials.2010.01.121

[cre270139-bib-0018] Gong, W. , Z. Huang , Y. Dong , et al. 2014. “Ionic Extraction of a Novel Nano‐Sized Bioactive Glass Enhances Differentiation and Mineralization of Human Dental Pulp Cells.” Journal of Endodontics 40, no. 1: 83–88. 24331996 10.1016/j.joen.2013.08.018

[cre270139-bib-0019] Gronthos, S. , M. Mankani , J. Brahim , P. G. Robey , and S. Shi . 2000. “Postnatal Human Dental Pulp Stem Cells (DPSCs) in Vitro and in Vivo.” Proceedings of the National Academy of Sciences of the United States of America 97, no. 25: 13625–13630. 11087820 10.1073/pnas.240309797 PMC17626

[cre270139-bib-0020] Gutiérrez, M. L. , J. Guevara , and L. A. Barrera . 2012. “Semi‐Automatic Grading System in Histologic and Immunohistochemistry Analysis to Evaluate in Vitro Chondrogenesis.” Universitas Scientiarum 17, no. 2: 167–178.

[cre270139-bib-0021] Hoemann, C. D. , H. El‐Gabalawy , and M. D. McKee . 2009. “In Vitro Osteogenesis Assays: Influence of the Primary Cell Source on Alkaline Phosphatase Activity and Mineralization.” Pathologie Biologie 57, no. 4: 318–323. 18842361 10.1016/j.patbio.2008.06.004

[cre270139-bib-0022] Huang, G. T. J. 2011. “Dental Pulp and Dentin Tissue Engineering and Regeneration–Advancement and Challenge.” Frontiers in Bioscience 3: 788–800. 10.2741/e286 PMC3289134 21196351

[cre270139-bib-0023] Huang, M. , R. G. Hill , and S. C. F. Rawlinson . 2016. “Strontium (Sr) Elicits Odontogenic Differentiation of Human Dental Pulp Stem Cells (hDPSCs): A Therapeutic Role for Sr in Dentine Repair?” Acta Biomaterialia 38: 201–211. 27131573 10.1016/j.actbio.2016.04.037

[cre270139-bib-0024] Karakuzu‐Ikizler, B. , P. Terzioğlu , Y. Basaran‐Elalmis , B. S. Tekerek , and S. Yücel . 2020. “Role of Magnesium and Aluminum Substitution on the Structural Properties and Bioactivity of Bioglasses Synthesized From Biogenic Silica.” Bioactive materials 5, no. 1: 66–73. 31989060 10.1016/j.bioactmat.2019.12.007 PMC6965208

[cre270139-bib-0025] Kavitha, R. , B. Subha , S. Shanmugam , and K. Ravichandran . 2014. “Synthesis and Invitro Characterisation of Lithium Doped Bioactive Glass Through Quick Alkali Sol‐Gel Method.” Int J Innov Res Sci Eng 2: 2347–3207.

[cre270139-bib-0026] Kermani, F. , S. Mollazadeh Beidokhti , F. Baino , Z. Gholamzadeh‐Virany , M. Mozafari , and S. Kargozar . 2020. “Strontium‐And Cobalt‐Doped Multicomponent Mesoporous Bioactive Glasses (MBGs) for Potential Use in Bone Tissue Engineering Applications.” Materials 13, no. 6: 1348. 32188165 10.3390/ma13061348 PMC7143072

[cre270139-bib-0027] Khorami, M. , S. Hesaraki , A. Behnamghader , H. Nazarian , and S. Shahrabi . 2011. “In Vitro Bioactivity and Biocompatibility of Lithium Substituted 45S5 Bioglass.” Materials Science and Engineering: C 31, no. 7: 1584–1592.

[cre270139-bib-0028] Meskher, H. , F. Sharifianjazi , K. Tavamaishvili , M. Irandoost , D. Nejadkoorki , and P. Makvandi . 2024. “Limitations, Challenges and Prospective Solutions for Bioactive Glasses‐Based Nanocomposites for Dental Applications: A Critical Review.” Journal of Dentistry 150: 105331. 39216818 10.1016/j.jdent.2024.105331

[cre270139-bib-0029] Moayeri, A. , M. Nazm Bojnordi , S. Haratizadeh , A. Esmaeilnejad‐Moghadam , R. Alizadeh , and H. Ghasemi Hamidabadi . 2017. “Transdifferentiation of Human Dental Pulp Stem Cells Into Oligoprogenitor Cells.” Basic and Clinical Neuroscience Journal 8, no. 5: 387–394. 10.18869/nirp.bcn.8.5.387 PMC5691170 29167725

[cre270139-bib-0030] Moghanian, A. , S. Firoozi , and M. Tahriri . 2017a. “Characterization, In Vitro Bioactivity and Biological Studies of Sol‐Gel Synthesized SrO Substituted 58S Bioactive Glass.” Ceramics International 43, no. 17: 14880–14890.

[cre270139-bib-0031] Moghanian, A. , S. Firoozi , and M. Tahriri . 2017b. “Synthesis and in Vitro Studies of Sol‐Gel Derived Lithium Substituted 58S Bioactive Glass.” Ceramics international 43, no. 15: 12835–12843.

[cre270139-bib-0032] Moghanian, A. , S. Firoozi , M. Tahriri , and A. Sedghi . 2018. “A Comparative Study on the In Vitro Formation of Hydroxyapatite, Cytotoxicity and Antibacterial Activity of 58S Bioactive Glass Substituted by Li and Sr.” Materials Science and Engineering: C 91: 349–360. 10.1016/j.msec.2018.05.058. 30033264

[cre270139-bib-0033] Moghanian, A. , A. Ghorbanoghli , M. Kazem‐Rostami , et al. 2020a. “Novel Antibacterial Cu/Mg‐Substituted 58S‐Bioglass: Synthesis, Characterization and Investigation of In Vitro Bioactivity.” International Journal of Applied Glass Science 11, no. 4: 685–698.

[cre270139-bib-0034] Moghanian, A. , R. Portillo‐Lara , E. Shirzaei Sani , H. Konisky , S. H. Bassir , and N. Annabi . 2020a. “Synthesis and Characterization of Osteoinductive Visible Light‐Activated Adhesive Composites With Antimicrobial Properties.” Journal of Tissue Engineering and Regenerative Medicine 14, no. 1: 66–81. 31850689 10.1002/term.2964 PMC6992487

[cre270139-bib-0035] Moghanian, A. , M. Zohourfazeli , M. H. Mahdi Tajer , Z. Miri , S. Hosseini , and A. Rashvand . 2021. “Preparation, Characterization and in Vitro Biological Response of Simultaneous Co‐Substitution of Zr+ 4/Sr+ 2 58S Bioactive Glass Powder.” Ceramics International 47, no. 17: 23762–23769.

[cre270139-bib-0036] Moghanian, A. , M. Zohourfazeli , and M. H. M. Tajer . 2020b. “The Effect of Zirconium Content on In Vitro Bioactivity, Biological Behavior and Antibacterial Activity of Sol‐Gel Derived 58S Bioactive Glass.” Journal of Non‐Crystalline Solids 546: 120262.

[cre270139-bib-0037] Moonesi Rad, R. , A. Z. Alshemary , Z. Evis , D. Keskin , K. Altunbaş , and A. Tezcaner . 2018. “Structural and Biological Assessment of Boron Doped Bioactive Glass Nanoparticles for Dental Tissue Applications.” Ceramics International 44, no. 8: 9854–9864.

[cre270139-bib-0038] Mozafari, M. , F. Moztarzadeh , and M. Tahriri . 2010. “Investigation of the Physico‐Chemical Reactivity of a Mesoporous Bioactive SiO_2_–CaO–P_2_O_5_ Glass in Simulated Body Fluid.” Journal of Non‐Crystalline Solids 356, no. 28–30: 1470–1478.

[cre270139-bib-0039] Nandi, S. K. , A. Mahato , B. Kundu , and P. Mukherjee . 2016. “Doped Bioactive Glass Materials in Bone Regeneration.” Advanced Techniques in Bone Regeneration 10: 63266.

[cre270139-bib-0040] Naruphontjirakul, P. , O. Tsigkou , S. Li , A. E. Porter , and J. R. Jones . 2019. “Human Mesenchymal Stem Cells Differentiate Into an Osteogenic Lineage in Presence of Strontium Containing Bioactive Glass Nanoparticles.” Acta Biomaterialia 90: 373–392. 30910622 10.1016/j.actbio.2019.03.038

[cre270139-bib-0041] Nazemi, Z. , M. Mehdikhani‐Nahrkhalaji , M. H. Nazarpak , and H. Staji . 2014. “Antibacterial Effect of Bioactive Glass Nanoparticles Prepared via Sol Gel Method.” Journal of Kermanshah University of Medical Sciences 18, no. 7: 381–387.

[cre270139-bib-0042] Omar, S. A. , J. Ballarre , Y. Castro , et al. 2020. “58S and 68S Sol‐Gel Glass‐Like Bioactive Coatings for Enhancing the Implant Performance of AZ91D Magnesium Alloy.” Surface and Coatings Technology 400: 126224.

[cre270139-bib-0043] Park, J. , G. E. Akbaba , N. Sharma , et al. 2025. “Electrically Active Biomaterials for Stimulation and Regeneration in Tissue Engineering.” Journal of biomedical materials research. Part A 113, no. 1: e37871. 39806919 10.1002/jbm.a.37871 PMC11773453

[cre270139-bib-0044] Pazhouheshgar, A. , A. Moghanian , and S. Sadough Vanini . 2020. “The Extended Finite Element Method Numerical and Experimental Analysis of Mechanical Behavior of Polysulfone/58s Bioactive Glass Synthesized Through Solvent Casting Method.” Modares Mechanical Engineering 20, no. 8: 2061–2073.

[cre270139-bib-0045] Pazhouheshgar, A. , S. A. Sadough Vanini , and A. Moghanian . 2019. “The Experimental and Numerical Study of Fracture Behavior of 58s Bioactive Glass/Polysulfone Composite Using the Extended Finite Elements Method.” Materials Research Express 6, no. 9: 095208.

[cre270139-bib-0046] Saatchi, A. , A. R. Arani , A. Moghanian , and M. Mozafari . 2021. “Synthesis and Characterization of Electrospun Cerium‐Doped Bioactive Glass/Chitosan/Polyethylene Oxide Composite Scaffolds for Tissue Engineering Applications.” Ceramics International 47, no. 1: 260–271.

[cre270139-bib-0047] Saleh, H. E.‐D. M. , and E. El‐Adham . 2018. *Trace Elements: Human Health and Environment*. BoD–Books on Demand.

[cre270139-bib-0048] Tang, L. , Y. Chen , F. Pei , and H. Zhang . 2015. “Lithium Chloride Modulates Adipogenesis and Osteogenesis of Human Bone Marrow‐Derived Mesenchymal Stem Cells.” Cellular Physiology and Biochemistry 37, no. 1: 143–152. 26303458 10.1159/000430340

[cre270139-bib-0049] Tavakolizadeh, A. , M. Ahmadian , M. H. Fathi , A. Doostmohammadi , E. Seyedjafari , and A. Ardeshirylajimi . 2017. “Investigation of Osteoinductive Effects of Different Compositions of Bioactive Glass Nanoparticles for Bone Tissue Engineering.” ASAIO Journal 63, no. 4: 512–517. 28033183 10.1097/MAT.0000000000000509

[cre270139-bib-0050] Vollenweider, M. , T. J. Brunner , S. Knecht , et al. 2007. “Remineralization of Human Dentin Using Ultrafine Bioactive Glass Particles.” Acta Biomaterialia 3, no. 6: 936–943. 17560183 10.1016/j.actbio.2007.04.003

[cre270139-bib-0051] Wu, X. , G. Meng , S. Wang , F. Wu , W. Huang , and Z. Gu . 2015. “Zn and Sr Incorporated 64S Bioglasses: Material Characterization, In‐Vitro Bioactivity and Mesenchymal Stem Cell Responses.” Materials Science and Engineering: C 52: 242–250. 25953564 10.1016/j.msec.2015.03.057

[cre270139-bib-0052] Yu, J. , Y. Wang , Z. Deng , et al. 2007. “Odontogenic Capability: Bone Marrow Stromal Stem Cells Versus Dental Pulp Stem Cells.” Biology of the Cell 99, no. 8: 465–474. 17371295 10.1042/BC20070013

[cre270139-bib-0053] Zamani, A. , G. R. Omrani , and M. M. Nasab . 2009. “Lithium's Effect on Bone Mineral Density.” Bone 44, no. 2: 331–334. 18992857 10.1016/j.bone.2008.10.001

[cre270139-bib-0054] Zhang, K. , A. Alaohali , N. Sawangboon , P. T. Sharpe , D. S. Brauer , and E. Gentleman . 2019. “A Comparison of Lithium‐Substituted Phosphate and Borate Bioactive Glasses for Mineralised Tissue Repair.” Dental Materials 35, no. 6: 919–927. 30975482 10.1016/j.dental.2019.03.008 PMC6559152

[cre270139-bib-0055] Zhang, M. , X. Zhang , J. Luo , et al. 2020. “Investigate the Odontogenic Differentiation and Dentin–Pulp Tissue Regeneration Potential of Neural Crest Cells.” Frontiers in Bioengineering and Biotechnology 8: 475. 32582651 10.3389/fbioe.2020.00475 PMC7290043

[cre270139-bib-0056] Zhao, X. , B. C. Heng , S. Xiong , et al. 2011. “In Vitro Assessment of Cellular Responses to Rod‐Shaped Hydroxyapatite Nanoparticles of Varying Lengths and Surface Areas.” Nanotoxicology 5, no. 2: 182–194. 21609137 10.3109/17435390.2010.503943

[cre270139-bib-0057] Zhong, J. , X. Tu , Y. Kong , et al. 2020. “LncRNA H19 Promotes Odontoblastic Differentiation of Human Dental Pulp Stem Cells by Regulating miR‐140‐5p and BMP‐2/FGF9.” Stem Cell Research & Therapy 11: 202. 32460893 10.1186/s13287-020-01698-4 PMC7251819

[cre270139-bib-0058] Zohourfazeli, M. , M. H. Mahdi Tajer , and A. Moghanian . 2021. “Comprehensive Investigation on Multifunctional Properties of Zirconium and Silver Co‐Substituted 58S Bioactive Glass.” Ceramics International 47, no. 2: 2499–2507.

